# Ultrasonic Non-Destructive Detection Method for Residual Stress in Rotary Forging Aluminum Alloy Plates

**DOI:** 10.3390/ma17112528

**Published:** 2024-05-24

**Authors:** Hongyu Chen, Xiaokai Wang, Xinghui Han, Fangyan Zheng, Wenlong Yan

**Affiliations:** 1 Hubei Key Laboratory of Advanced Technology for Automotive Components, Wuhan University of Technology, Wuhan 430070, China; hongyuchen1799@whut.edu.cn (H.C.); hanxinghuihlp@126.com (X.H.); 13013@whut.edu.cn (F.Z.); ywl0922@whut.edu.cn (W.Y.); 2 Hubei Collaborative Innovation Center for Automotive Components Technology, Wuhan University of Technology, Wuhan 430070, China; 3 School of Automotive Engineering, Wuhan University of Technology, Wuhan 430070, China

**Keywords:** aluminum alloy plate, residual stress, longitudinal critically refracted (LCR) wave, one-transmitter and double-receiver (OTDR) transducer, rotary forging

## Abstract

Aluminum alloy plates are widely used to manufacture large-scale integral structure parts in the field of aerospace. During the forming and processing of aluminum alloy plates, different degrees of residual stress are inevitably produced. Fast and accurate detection of residual stress is very essential to ensuring the quality of these plates. In this work, the longitudinal critically refracted (LCR) wave detection method based on a one-transmitter and double-receiver (OTDR) transducer and the finite element simulation were employed to obtain the residual stress. Aluminum alloy plates with different deformation amounts were fabricated by rotary forging to obtain different residual stress states. Results reveal that the plate formed by rotary forging is in a stress state of central tension and edge compression. As the deformation increases from 20% to 60%, the peak residual tensile stress increases from 156 MPa to 262 MPa, and there is no significant difference in the peak compressive stress. When the deformation reaches 60%, the difference in the residual stresses at different depths is less than 13%, which indicates that the plastic deformation zone basically penetrates the entire longitudinal cross-section of the plate. The maximum deviation between measurement and FE is 61 MPa, which means the experimental data are in good agreement with the FE results.

## 1. Introduction

The aerospace industry extensively employs high-performance aluminum alloy plates due to their advantageous features, including lightweight properties, exceptional strength, and remarkable load-bearing capacity [1]. These plates are crucial in the production of vital components such as aircraft fuselages, rocket bodies, and space station cabins [2,3]. Plastic forming is capable of effectively refining the grain structure of metal materials and is an important manufacturing technology for high-performance aluminum alloy plate parts [4]. However, the metal-material plastic forming process will produce non-uniform plastic deformation, resulting in residual stresses [5,6,7]. The existence of residual stress has a remarkable impact on the strength and service life of components [8]. Residual stress detection of plastic-formed aluminum alloy plates holds immense importance, particularly for aerospace components that endure extreme conditions such as high temperatures, heavy loads, cyclic loads, and others [9]. The residual stress can also potentially lead to brittle failure and stress corrosion cracking, thereby posing a significant risk to safety [10].

Previous research has proposed three types of residual stress detection methods: destructive detection, half-destructive detection, and non-destructive detection [11]. The half-destructive and destructive methods, also named the mechanical detection method, include the ring core, blind hole, and deep hole methods. Its principle is to calculate the residual stress through the displacement generated when the stress is completely or partially released during the material removal process. Non-destructive detection usually includes the diffraction method, magnetic method, and ultrasonic method, which typically measure physical parameters related to stress and thus calculate the value of residual stress. Among the detection methods mentioned above, ultrasonic residual stress detection has many advantages, including but not limited to fast detection speed, no radiation damage, good spatial resolution, and a deep detection range [12]. In addition, the ultrasonic testing method can detect surface and sub-surface residual stress and evaluate the tensile and compressive states of components. Bray [13] was the first scholar to propose the use of a longitudinal critically refracted (LCR) wave to detect longitudinal residual stress in materials and put it into effect in welded components, obtaining relatively reliable data. Javadi [14] employed the LCR wave to detect friction stir-welded samples, and the experimental data were compared with the drilling method, which has a good agreement. Subsequently, these researchers utilized finite-element simulation to assess the welding residual stress in dissimilar joints and compared immersion and contact ultrasonic techniques [15]. Furthermore, they detected the residual stresses in different depths of welded plates and welded pipes, respectively, using LCR waves [16,17,18]. Cao [19] detected the residual stress of 7050 aluminum alloy plates with different thicknesses using ultrasonic technology and illustrated that the residual stress of the plates after aging was at a lower level.

However, the detection systems used in these studies are complex and cannot detect workpieces in service. Furthermore, the performance of the acquisition card needs to be improved so as to enhance the accuracy of acoustic time measurement. In addition, the research on rotary forging for aluminum alloy plate parts mainly focuses on the process design, while the research on the distribution of residual stress and detection methods is rarely reported.

To solve these issues, an ultrasonic evaluation model of residual stress based on a one-transmitter and double-receiver (OTDR) transducer was established in this paper. Three sets of OTDR transducers with a center frequency of 2.5/5/10 MHz were designed and fabricated. A portable residual stress device with a high sampling rate of 5 GS/s has been designed and manufactured, which can be used for the detection of parts in service. Automatic detection of residual stresses is realized based on this device and a collaborative machine with force sensors. Then, the residual stress detection was completed on aluminum alloy plates formed by rotary forging. To increase the detection accuracy, a stress coefficient calibration experiment was conducted for the OTDR transducer. Finally, a comparative analysis was conducted between the detection data and the finite element simulation data.

## 2. Theoretical Background

### 2.1. The Theory of Longitudinal Critically Refracted Wave Detection

When the ultrasonic longitudinal wave travels from medium I to medium II at the first critical angle, the LCR wave propagating parallel to the surface of medium II could be generated [20]. Under the assumption of uniform deformation and solid isotropy, the relationship between ultrasonic velocity *V* and stress *σ* can be obtained on the basis of acoustoelastic theory. When the direction of the longitudinal wave is in accordance with the direction of stress, *V* and *σ* are related by [21]:

(1) $$\rho_{0}V^{2}=\lambda +2\mu +\frac{\sigma }{3\lambda +2\mu }\left[\frac{\lambda +\mu }{\mu }(4\lambda +10\mu +4m)+\lambda +2l\right]$$

where $\rho_{0}$ is the density of a zero-stress sample; *V* is the velocity of the longitudinal wave whose direction of propagation is in line with the direction of stress; *λ*, *μ*, *l*, *m*, and *n* are elastic constants; and *σ* is the value of stress, which indicates compressive stress when it is negative and tensile stress when it is positive.

**Figure 1:**
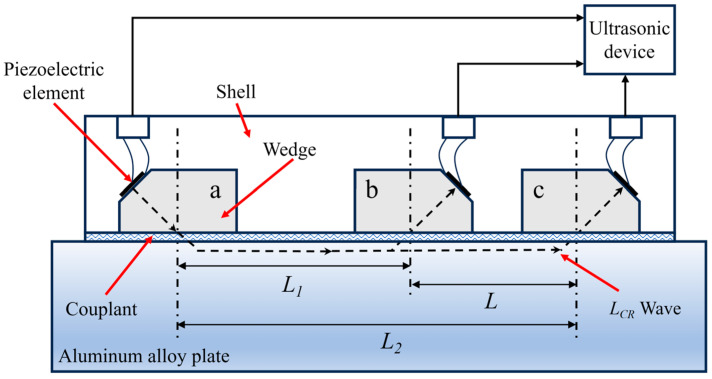
Schematic diagram of the OTDR transducer.

In this experiment, the distance *L* between transducers is a constant; therefore, the change in velocity can be reflected by calculating the variation in the propagation time of ultrasonics (PTOU). The calculation formula for residual stress detection of LCR waves is shown as follows:

(2) Δσ=KΔt

(3) $$K=\frac{-2V_{0}\left(3\lambda +2\mu \right)}{\left(\frac{4\lambda +10\mu +4m}{\mu }+\frac{2l-3\lambda -10\mu -4m}{\lambda +2\mu }\right)L}$$

where *K* is known as the acoustoelastic coefficient; *V_0_* is the velocity of ultrasonic in a zero-stress sample; Δσ is the stress of the workpiece being detected; and Δt is the variation in the PTOU between the workpiece and the zero-stress sample. Figure 1 is the schematic diagram of the OTDR probe. *L* is the distance between receivers b and c, and then the time delay $t_{D}$ of two receivers can be obtained as follows:

(4) $$t_{D}=\left(t_{a}^{\prime }+t_{a}+t_{2}+t_{c}+t_{c}^{\prime }\right)-\left(t_{a}^{\prime }+t_{a}+t_{1}+t_{b}+t_{b}^{\prime }\right)=t_{2}-t_{1}+\left(t_{c}^{\prime }-t_{b}^{\prime }\right)+\left(t_{c}-t_{b}\right)$$

where $t_{a}^{\prime }$, $t_{b}^{\prime }$, $t_{c}^{\prime }$ is the PTOU in wedges *a*, *b*, *c*, respectively; $t_{a}^{\prime \prime }$, $t_{b}^{\prime \prime }$, $t_{c}^{\prime \prime }$ is the PTOU in couplant under the three wedges, respectively; and $t_{1}$, $t_{2}$ is the PTOU in material for distances *L_1_* and *L_2_*, respectively. The materials and dimensions of the three wedges are the same, so it can be considered that the PTOU in the three wedges is equal. A mechanical arm with a force sensor is used to control the pressing force of the probe, thus ensuring the consistency of coupling conditions. Therefore, the PTOU in the coupling layer under the wedges is approximately equal. Then Formula (4) can be simplified as follows:

(5) $$t_{D}=t_{2}-t_{1}$$

The exact PTOU in the material can then be obtained. In addition, wedges with the same geometric shape compensate for the influence of temperature on the PTOU, which is beneficial for improving detection accuracy.

### 2.2. Principles of the LCR Wave Method for Detecting Residual Stress at Different Depths

The penetration depth of the LCR wave varies with the center frequency, and the following empirical formula reveals this law [22]:

(6) $$D=\alpha_{S}f^{-0.96}$$

where *D* is the penetration depth (mm); $\alpha_{S}$ is measured by the experiment (6.4 for aluminum alloy); and *f* is the frequency of the probe. As exhibited in Table 1, according to Formula (6), the penetration depths of probes (2.5/5/10 MHz) in 6061 aluminum alloy were calculated.

## 3. Sample Preparation and Finite Element Modeling

### 3.1. Sample Preparation

**Figure 2:**
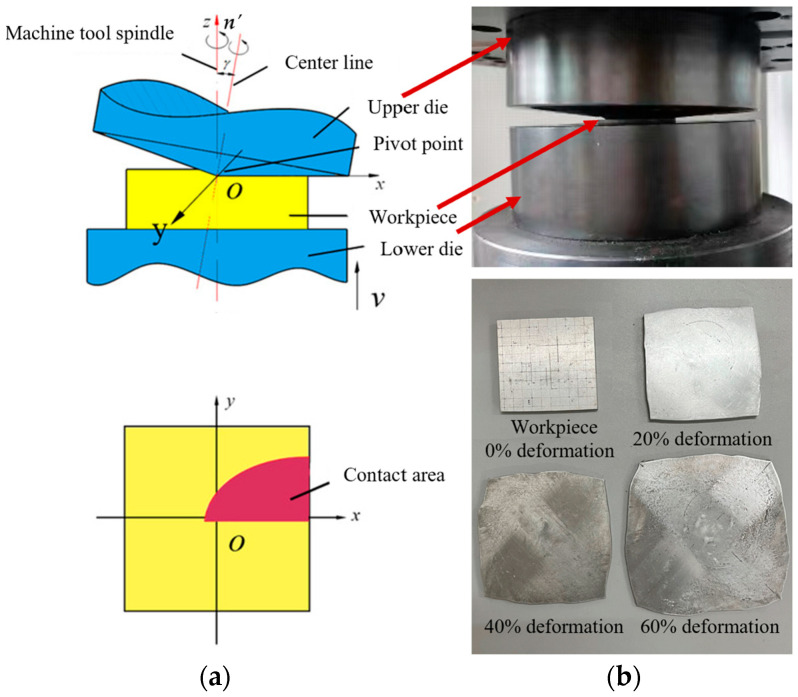
Sample preparation [25]: (**a**) principle of rotary forging and (**b**) equipment and sample.

Aluminum alloy plates with different deformations are manufactured using rotary forging, as exhibited in Figure 2. The upper die, which is conical in shape and inclined at an angle *γ*, rotates around the center line and the machine tool spindle, respectively. The lower die moves upward at a certain feed speed to exert pressure on the workpiece. Under the pressing of the upper and lower dies, continuous local plastic deformation accumulates in the workpiece to achieve integral plastic formation [23,24]. Rotary forging is a multi-degree-of-freedom machining process, and the deformation mechanism of the workpiece is relatively complex. Therefore, a reasonable selection of process parameters is particularly important. For the processing of aluminum alloy plates, the upper die carries out the conventional circular trajectory movement. In this case, the inclination angle of the upper die, the oscillating speed of the upper die, the feed rate of the lower die, and the friction factor are the key parameters. Detailed process parameters are given in Table 2. The material used in this study is a 6061 aluminum alloy with a tensile strength of 310 MPa and a yield strength of 285 MPa. The size of the aluminum alloy plate blank is 10 × 10 × 10 mm^3^, and the deformation increases by 10% for each 1 mm increase in the amount of feed exerted by the lower die. Finally, aluminum alloy plates with different deformation amounts (20%, 40%, and 60%) were obtained.

### 3.2. FE Model for Simulating Rotary Forging

The finite element simulation of the rotary forging was conducted by Deform-3D software (https://www.deform.com/products/deform-3d/). The simulation parameters are the same as the machining parameters in Table 2. The plate blank is designed as a square with a side length of 100 mm and a thickness of 10 mm. The material of the plate blank is 6061 aluminum alloy, and it is set as an elastic-plastic body. Both the upper and lower dies are set as rigid bodies, and the material is forged die steel. The plate blank is discreted by a tetrahedral mesh. After unloading, constraint transformation, and cooling [26], residual stress distribution maps of aluminum alloy plates formed by rotary forging were obtained.

**Figure 3:**
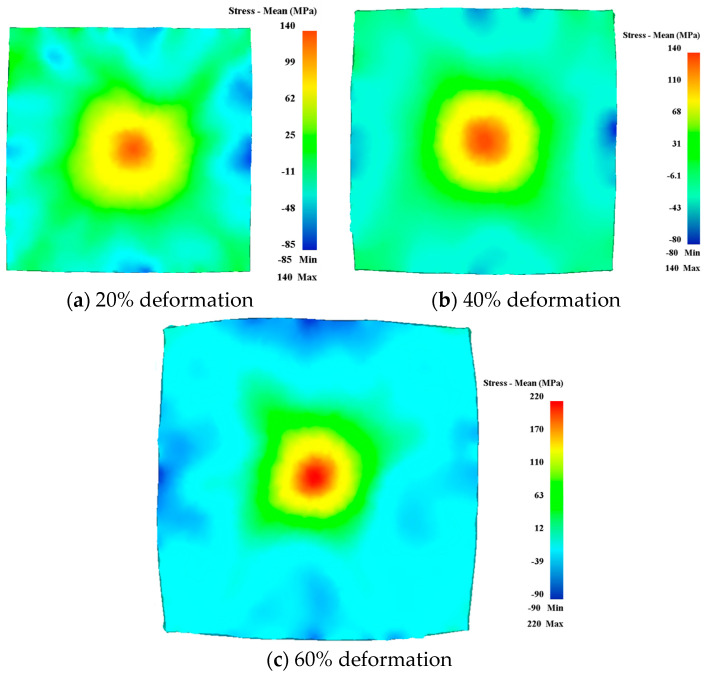
Residual stress distribution maps: (**a**) 20% deformation, (**b**) 40% deformation, and (**c**) 60% deformation.

Figure 3 illustrates that the residual stress of the rotary forging plate is approximately distributed in a central symmetry. The maximum and minimum values of stress appear at the center and edge of the workpiece, respectively. From the center to the edge position, the residual stress decreases gradually and changes from tensile stress to compressive stress. Furthermore, as the deformation increases from 20% to 60%, the stress values increase obviously, but the distribution of stress does not vary significantly. The results obtained from the analysis are in conformance with the characteristics of rotary forging.

## 4. Experimental Procedures

### 4.1. LCR Wave Residual Stress Ultrasonic Detecting System

The residual stress ultrasonic non-destructive detecting system mainly includes a data acquisition board, ultrasonic transducers, an IPC (industrial personal computer) with corresponding algorithm software, communication systems, and an automatic scan device. The data acquisition board has a high sampling rate of 5 GS/s and an ultrasonic time measurement accuracy of 0.2 ns. The ultrasonic transducer uses the OTDR mode.

### 4.2. Design of OTDR Transducers

For the purpose of detecting and obtaining the residual stress distribution along the depth direction of workpieces with different deformations, it is necessary to manufacture OTDR transducers with different frequencies.

The wedges are made of organic glasses, the piezoelectric element is 6 mm in diameter, and the center frequency is 2.5/5/10 MHz, respectively. Snell’s law provides a method for calculating the first critical angle, and then the angle of wedges is calculated as 25.6°. Considering that using high-frequency transducers can cause significant signal attenuation and affect the signal-to-noise ratio if the propagation distance is too long, the distances between the transmitter and the two receivers are designed to be 20 mm and 40 mm, respectively.

### 4.3. Verifying the Design with the FE Model

**Figure 4:**
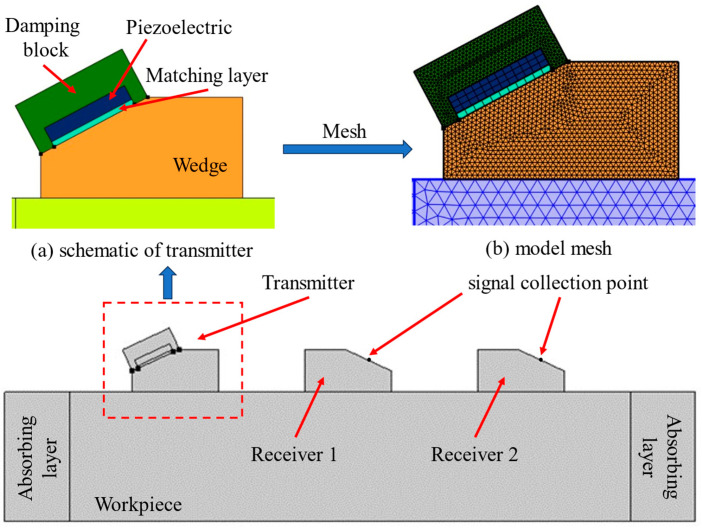
Simulation model: (**a**) schematic of the transmitter and (**b**) model mesh.

Acoustic simulation of the OTDR transducer was performed by COMSOL software (https://cn.comsol.com/) to verify the feasibility of the design parameters. The simulation models and meshing are displayed in Figure 4. The unit is driven by a voltage signal applied to the piezoelectric transducer, and the center frequency of the signal is 2.5 MHz. The piezoelectric transducer is attached to the wedge via a matching layer and surrounded by a damping block at the back. The piezoelectric transducer and the matching layer are half and one-quarter wavelength thick, respectively. The matching layer and the damping block materials should be chosen in such a way that the acoustic impedance of the former is close to that of the transducer, while the impedance of the latter is close to the geometric mean of those of the transducer and the wedge. Typical materials used for the matching and backing layers are alumina/epoxy or tungsten/epoxy composites. The properties of the components used in this model are listed in Table 3.

The model geometry is an assembly, which makes the parts of the geometry separate from one another and connected via identity boundary pairs. Each physics interface automatically imposes the continuity boundary condition on all boundary pairs. For wave propagation problems, feasible results are achieved when the mesh resolves the wavelengths of the propagating waves. The wavelength, in turn, depends on the speed of sound in the material. Thus, materials with a lower speed of sound require a finer mesh than those with a higher speed of sound. In the mesh toolbar, select mapped to mesh the piezoelectric transducer and the matching layer. The rest of the parts are meshed by free triangles, and all the mesh sizes meet Formula (7):

(7) $$d\leq \frac{C_{L}}{3f_{S}}$$

where *d* is the maximum mesh size (m); *C_L_* is the longitudinal wave velocity of material (m/s); and *f_S_* is the frequency of the excitation signal (MHz).

**Figure 5:**
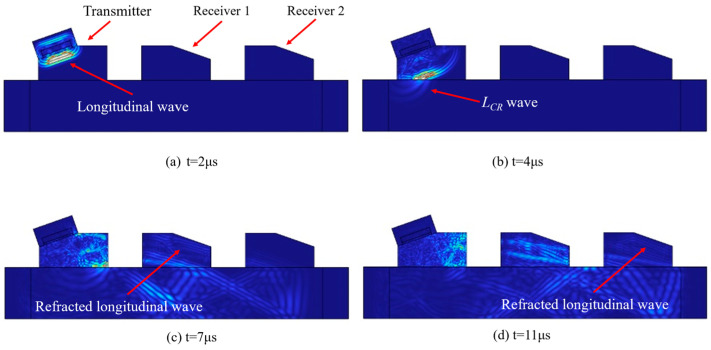
Acoustic simulation of the OTDR transducer.

Figure 5 shows the evolution of the ultrasonic signal. When t = 2 μs, the transmitter transducer completes the emission of the excitation signal, and the longitudinal wave is generated in the wedge. When t = 4 μs, the longitudinal wave refracts at the interface between the wedge and the sample, generating an LCR wave. When t = 7 μs and t = 11 μs, the LCR wave refracts again and reaches the signal collection point of the first and second receiver transducers, respectively.

**Figure 6:**
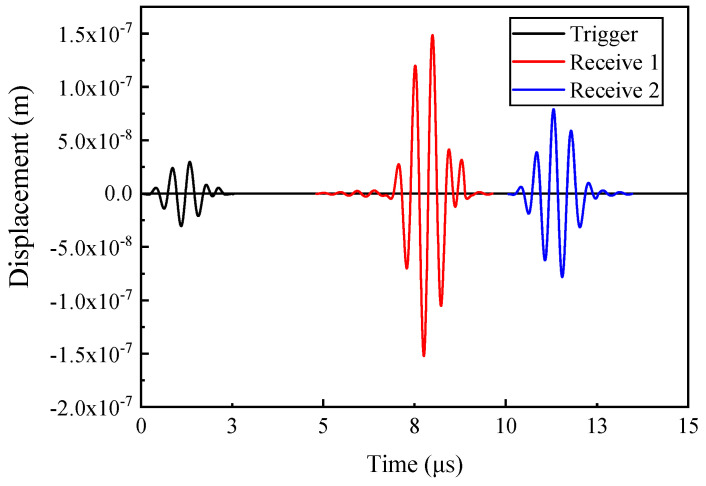
Displacement signal.

Figure 6 illustrates the displacement signal collected by the detection point. It is apparent that the signal waveform received by the two receivers is similar. The speed of the LCR wave in aluminum alloy calculated based on these two waves is 6205 m/s, which is in accordance with the theoretical value. Due to the increased propagation distance, the signal received by the second receiver is weaker than that of the first one, which explains why the distance of transducers cannot be designed to be too long.

### 4.4. Design of the Detection Device

**Figure 7:**
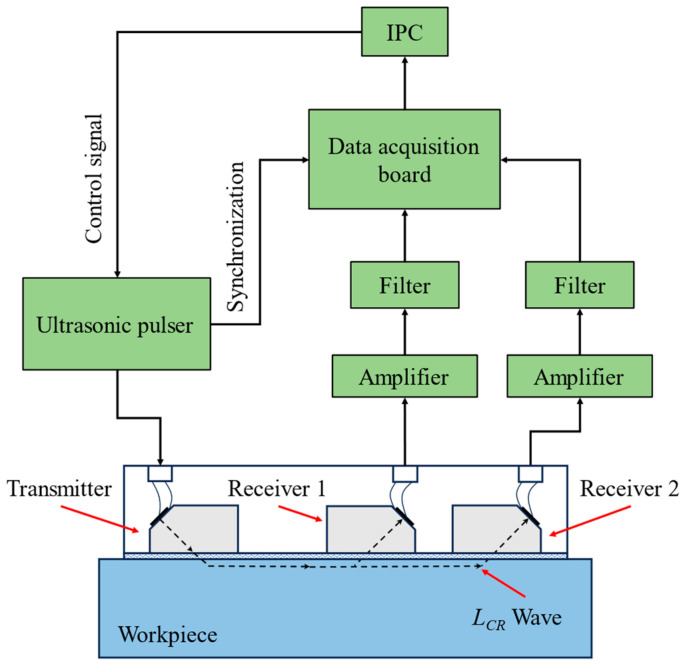
Schematic of the detection device.

The residual stress detection device was designed according to the signal processing flow. As shown in Figure 7, the transmitter transducer emits an ultrasonic testing signal under the control of the ultrasonic pulser. Two receiver transducers receive the ultrasonic signal and convert it into an electrical signal. The analog signal is processed through amplifier and filter circuits, then converted into a digital signal by the data acquisition board. The IPC receives a signal from the data acquisition board and sends a control command to the ultrasonic pulser.

**Figure 8:**
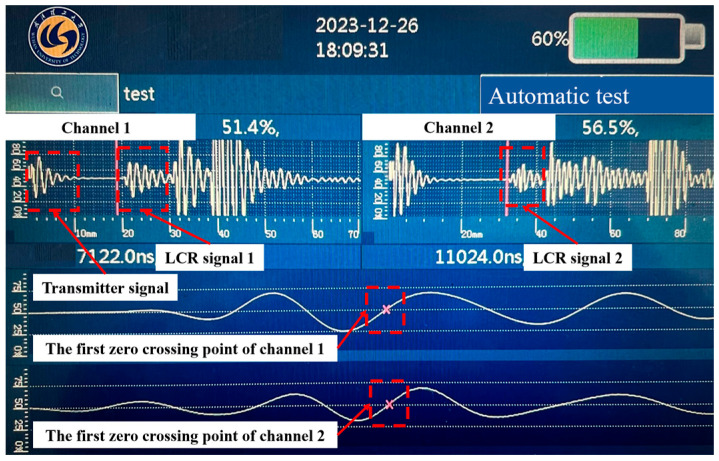
Display interface of the ultrasonic residual stress detection device.

The display interface of the ultrasonic residual stress detection device is exhibited in Figure 8. Channels 1 and 2 display the ultrasonic signals received by two receiver transducers, respectively. Taking channel 1 as an example, the first group of waveforms is the signal that is emitted by the transmitter transducer, and the second group of waveforms is the ultrasonic signal that first reaches the receiving transducer. In view of the fact that the LCR wave is a longitudinal wave, whose propagation speed is the fastest and propagation path is the shortest, the second group of waveforms mentioned above is the LCR wave signal. The zero-crossing algorithm is used to calculate the PTOU. When the amplitude of the signal received by the ultrasonic transducer exceeds the set threshold (70% in this paper), the zero-crossing detection circuit is opened. By capturing the first zero-crossing point before the threshold, the PTOU in the current channel can be determined. The acoustic time acquired by the two channels can be used to calculate the value of residual stress.

### 4.5. Calibration Experiment

**Figure 9:**
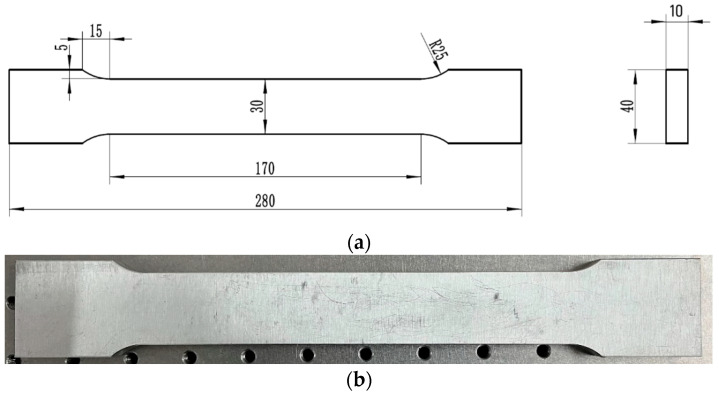
The test specimens: (**a**) size and (**b**) specimen.

To improve detection accuracy, it is necessary to calibrate the detection system by using the universal testing machine in a laboratory environment before detecting residual stress. The error of the detection system is determined based on the standard stress value provided by the universal testing machine. Before the tensile test, zero-stress specimens need to be prepared. Therefore, an annealing treatment was carried out to release residual stress in the specimens. Figure 9 shows the specimen used in this study and its drawing size.

**Figure 10:**
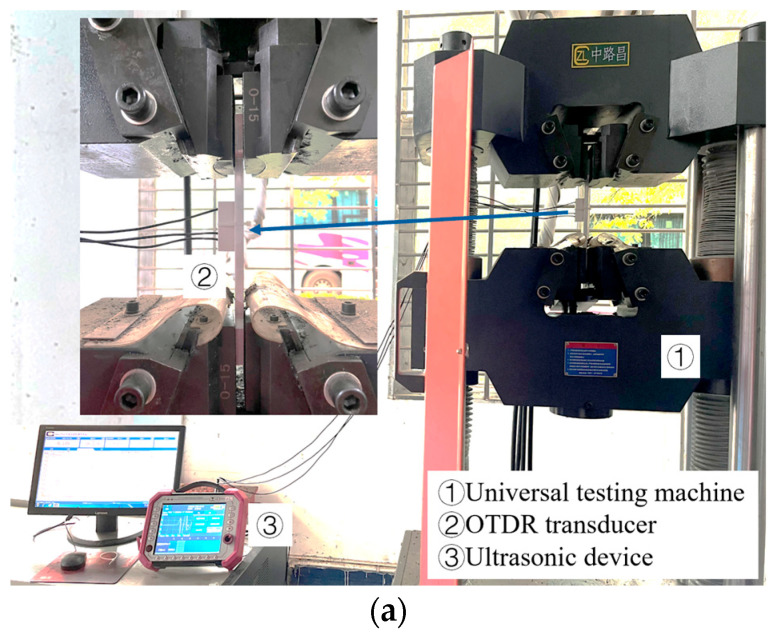

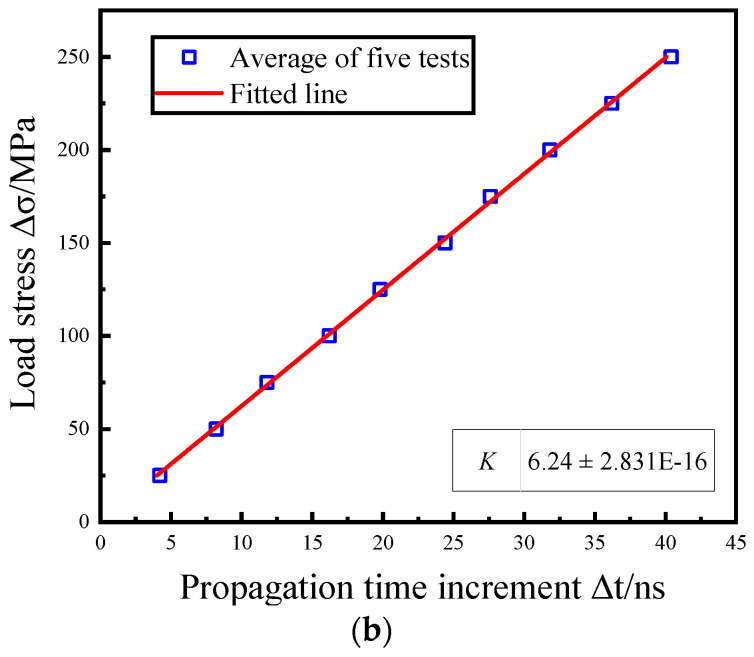
Stress coefficient calibration test: (**a**) test devices and (**b**) data.

The tensile test was conducted following the methodology outlined in ISO 6892-1:2009, maintaining an ambient temperature of 22 ± 2 °C [27]. In the elastic range of aluminum alloy, ten test points were uniformly selected to complete the tensile test. The stress was increased by 25 MPa each time, and the propagation time of the LCR wave in each stress state was recorded. The tests mentioned above were repeated 5 times, and the relationship between stress increment Δ*σ* and time increment Δ*t* was obtained after average and linear fitting of the data [28]. As shown in Figure 10b, the *K* (stress coefficient) of 6061 aluminum alloy is 6.24 (MPa/ns) when the distance is *L* = 20 mm, which is consistent with the theoretical calculation value.

### 4.6. Residual Stress Detection Method

**Figure 11:**
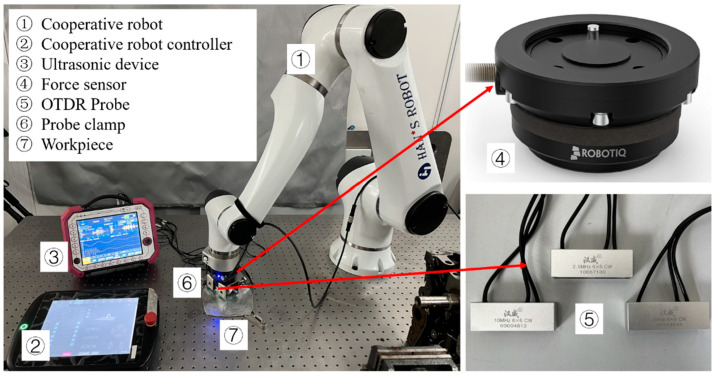
Schematic diagram of the detection method.

Figure 11 illustrates both the detection system and the experimental method. On the basis of the forming characteristics of aluminum alloy plates fabricated by rotary forging, detecting positions were uniformly selected (every 5 mm) along the diagonal direction of the plate, and probes were placed at these positions to measure residual stress values. The detection depth was changed by replacing probes with different center frequencies. The probe is fixed on the mechanical arm with a clamp, and the mechanical arm moves along the diagonal direction (main stress direction) of the plate. The force sensor is used to detect the pressing force in real time to ensure that the coupling conditions at each detection position are consistent. A medical ultrasonic couplant is used for coupling.

## 5. Results and Discussions

### 5.1. Effect of Deformation on Residual Stress

**Figure 12:**
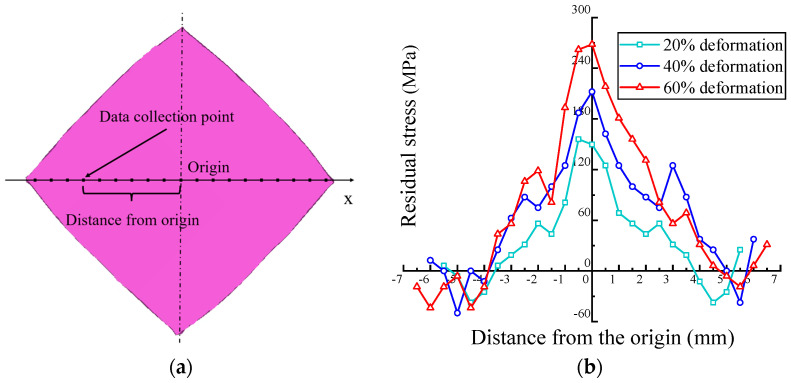
Residual stress of a plate with different deformations: (**a**) coordinates and (**b**) data.

In order to analyze the relationship between deformation and residual stresses, the OTDR transducer (2.5 MHz) was used to detect aluminum alloy plates with different deformations. Figure 12a shows the coordinates, and Figure 12b illustrates that the maximum stress appears at the center position of the workpiece. From the center to the edge position, the residual stress decreases accordingly until it reaches the minimum value and then increases insignificantly. As the deformation increases from 20% to 60%, the peak residual tensile stress increases from 156 MPa to 262 MPa, showing an increase of 68%. Furthermore, there is no significant difference in the peak compressive stress, which is around −40 MPa. The results are in agreement with the forming mechanism in previous studies, that is, plastic deformation first occurs in the center area of contact between the plate and the upper die, and the plastic deformation area gradually expands to the edge of the plate with the rotation of the upper die [29].

### 5.2. Comparison of the FE and Test Results

**Figure 13:**
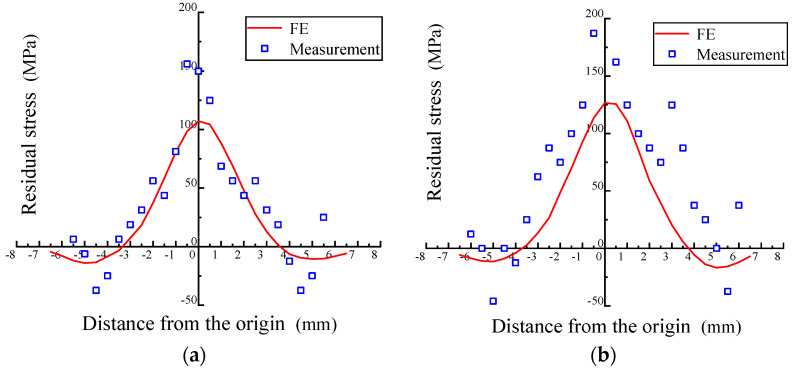

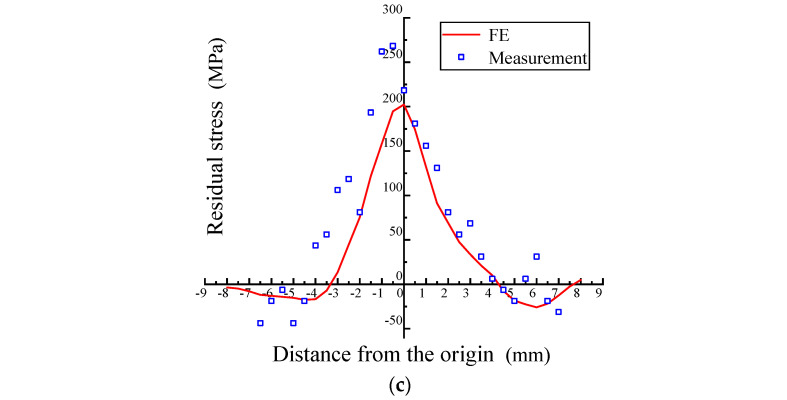
Comparison between the FE and measurement (2.5 MHz): (**a**) 20% deformation, (**b**) 40% deformation, and (**c**) 60% deformation.

Test data were compared with FE results, as shown in Figure 13. When the deformation amount is 20%, 40%, or 60%, the maximum deviations between measurement and FE are 48 MPa, 61 MPa, and 59 MPa, respectively. The maximum deviation occurred near the plate center. The residual stress distribution pattern of the rotary forging plate obtained from detection is basically consistent with simulation results, which verifies the feasibility of the detection system.

### 5.3. Distribution of the Residual Stress at Different Depths

**Figure 14:**
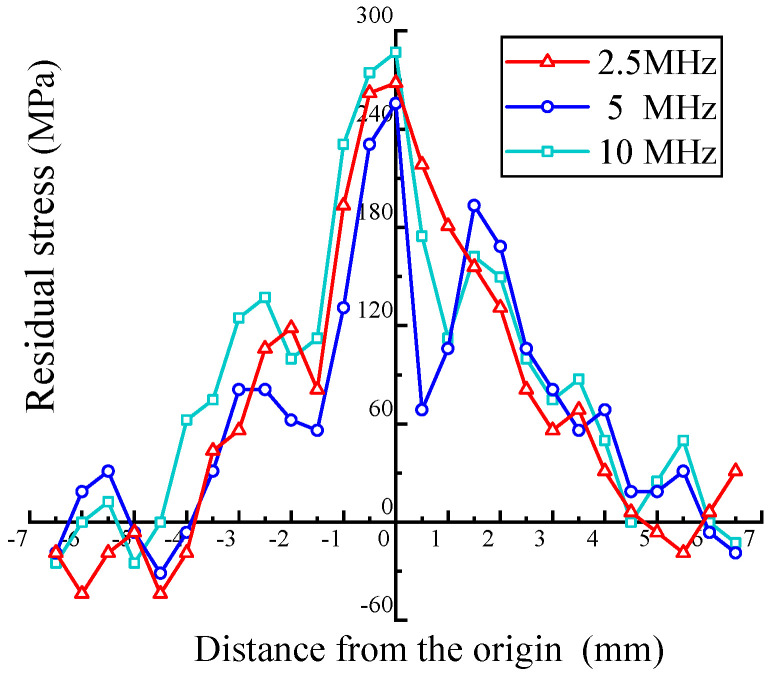
Residual stress distribution of a plate (60% deformation) at different depths.

For obtaining the residual stress in the depth direction, an aluminum alloy plate with 60% deformation was selected, and OTDR transducers with center frequencies of 2.5 MHz, 5 MHz, and 10 MHz were used to detect the stress at different depths. Figure 14 compares the results. When the deformation reaches 60%, the difference in the residual stresses at different depths is less than 13%. The reason for this result is that when the deformation reaches a high level, the plastic deformation zone basically penetrates the entire longitudinal cross-section of the aluminum alloy plate [30].

### 5.4. Residual Stress after Annealing

**Figure 15:**
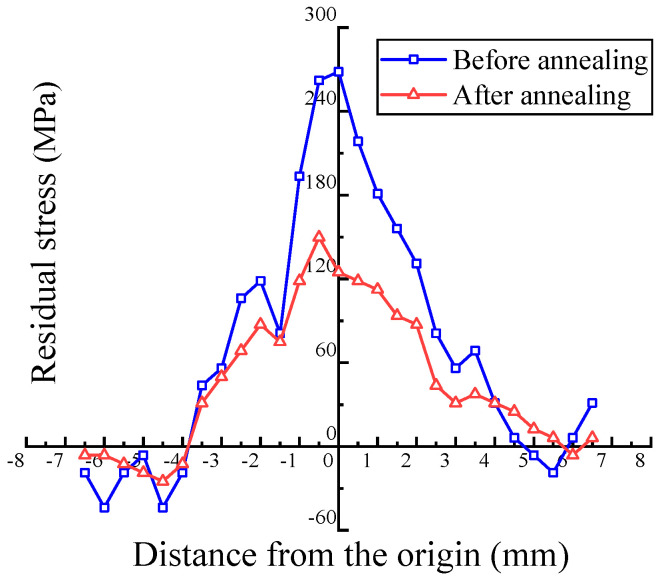
Contrast analysis of a 60% deformed aluminum alloy plate before and after stress relief annealing.

Based on the previous analysis, the value of residual stress in the plate increases with the amount of deformation. The workpiece bears a large residual tensile stress, especially in the center position. The macroscopic deformation pattern of the plate during the rotary forging is very complex. The metal in the center region of the plate is subjected to linearly increasing periodic tensile strain along the periphery in a radial manner and to linearly increasing compressive strain along the normal direction. This complex deformation pattern leads to a complex change in deformation stress in the center region of the plate during the forming process. Once the stress reaches a critical value, it results in the emergence and coalescence of cracks in the center layer, which rapidly extend to other regions, leading to overall cracking failure [31]. Therefore, aluminum alloy plates need to be treated with residual stress relief after rotary forging to avoid fatigue failure. The plate with 60% deformation was selected for residual stress detection after relief annealing. The results are shown in Figure 15. After relief annealing, the peak value of residual stress decreases by about 50%, and the stress distribution becomes smoother, which means the risk of fatigue cracking at the center position is significantly reduced.

## 6. Conclusions

Aiming at evaluating the residual stress of a rotary forging plate, finite element simulation and the ultrasonic method were employed in this paper. The main conclusions include the following:

(1) The experimental results show that the residual stress of the rotary forging plate (6061 aluminum alloy) has an approximately centrosymmetric distribution, and the maximum and minimum values of stress appear at the center and edge of the workpiece, respectively. As the deformation increases from 20% to 60%, the peak residual tensile stress increases from 156 MPa to 262 MPa, and there is no significant difference in the peak compressive stress, which is around −40 MPa. The maximum deviation between measurement and FE is 61 MPa, which means the experimental data are similar to the FE results.
(2) Transducers with frequencies of 2.5 MHz, 5 MHz, and 10 MHz were applied to obtain the residual stress distribution at different depths. When the deformation of the rotary forging plate reaches 60%, the difference in the residual stresses at different depths is less than 13%, which indicates that the plastic deformation zone basically penetrates the entire longitudinal cross-section of the aluminum alloy plate.
(3) The peak value at the center of the plate after stress relief annealing is reduced by about 50% compared with that before, and the stress distribution becomes smoother, which means the risk of fatigue cracking at the center position is significantly reduced.

## Figures and Tables

**Table 1:** Ultrasonic penetration depth of probes (aluminum alloy).

Center Frequencies (MHz)	2.5	5	10
Penetration Depth (mm)	2.7	1.4	0.7

**Table 2:** Process parameters of rotary forging.

Parameters	Values
Inclination angle of the upper die	2°
Oscillating speed of the upper die	240 r/min
Feed rate of the lower die	1 mm/s
Friction factor between the dies and workpiece	0.14

**Table 3:** Properties of the components.

Part	Material	Density (kg/m^3^)	Longitudinal Wave Speed (m/s)	Shear Wave Speed (m/s)
Piezoelectric	PZT-5H	7500	4620	1750
Wedge	Acrylic plastic	1190	2080	1000
Matching layer	Alumina/Epoxy	2280	3400	1920
Damping block	Tungsten/Epoxy	6580	1500	775

## Data Availability

This study does not use other people’s research data, and the data in this paper are all obtained through experiments.

## Abbreviations

**Symbol**	**Notation**
$\rho_{0}$	The density of material under zero-stress
*V*	The velocity of the longitudinal wave
*λ*, *μ*	The Lame elastic constants
*l*, *m*, *n*	The Murnaghan elastic constants
Δσ	The stress of the workpiece being detected
*K*	The acoustoelastic coefficient
Δt	The time variation under the condition of stress
*L*	The distance between two receivers
*L_1_*	The distance between the transmitter and the first receiver
*L_2_*	The distance between the transmitter and the second receiver
$t_{D}$	The time delay of two receivers
$t_{a}^{\prime }$	The acoustic time in wedge a
$t_{b}^{\prime }$	The acoustic time in wedge b
$t_{c}^{\prime }$	The acoustic time in wedge c
$t_{a}^{\prime \prime }$	The acoustic time in couplant under wedge a
$t_{b}^{\prime \prime }$	The acoustic time in couplant under wedge b
$t_{c}^{\prime \prime }$	The acoustic time in couplant under wedge c
$t_{1}$	The acoustic time at distance *L_1_*
$t_{2}$	The acoustic time at distance *L_2_*
*D*	The penetration depth
$\alpha_{S}$	The coefficient obtained by the experiment
*f*	The frequency of the probe
*γ*	The inclination angle of the upper die
*n’*	The oscillating speed of the upper die
*v*	The feed rate of the lower die
*d*	The maximum mesh size
*C_L_*	The longitudinal wave velocity of material
*f_S_*	The frequency of the excitation signal
